# Electrocatalytic CO_2_ fixation by regenerating reduced cofactor NADH during Calvin Cycle using glassy carbon electrode

**DOI:** 10.1371/journal.pone.0239340

**Published:** 2020-09-17

**Authors:** Irshad Ali, Saeid Amiri, Nehar Ullah, Mohammad Younas, Mashallah Rezakazemi

**Affiliations:** 1 Department of Chemical Engineering, University of Engineering & Technology, Peshawar, Pakistan; 2 Chemical & Petroleum Engineering Department, Sharif University of Technology, Tehran, Iran; 3 Faculty of Chemical and Materials Engineering, Shahrood University of Technology, Shahrood, Iran; Missouri University of Science and Technology, UNITED STATES

## Abstract

In this study, an enzymatic pathway has been developed to replicate the Calvin Cycle by creating the individual steps of the carbon cycle in a bioreactor. The technology known as “artificial photosynthesis” converts CO_2_ emissions into a variety of intermediates that serve as precursors to high-value products. CO_2_, light, water, and electricity were used as feedstock. An electrochemical reactor was also studied for the regeneration of active NADH operating at constant electrode potential. Initially, a batch electrochemical reactor containing 80 mL of 0.2 mM NAD^+^ in Tris-buffer (pH 7.40) was used to evaluate the electrode material operating at normal temperature and pressure. The results showed that the cathode is highly electrocatalytically efficient and selective to regenerate 97.45±0.8% of NADH from NAD^+^ at electrode potential of -2.3 V *vs*. mercury standard electrode (MSE). The NADH regeneration system was then integrated with ATP regeneration system and bioreactor containing Ribulose bisphosphate carboxylase/oxygenase (RuBisCO). NADH was regenerated successfully during the process electrochemically and then was used by the enzymatic reaction to produce triose phosphate and 3-Phosphoglycerate (3GPA).

## Introduction

Economic growth along with high density infrastructural and transportation development is mainly responsible for rapid energy demand that consequently resulted in an increased emission of carbon dioxide (CO_2_). CO_2_ is the primary anthropogenic greenhouse gas (GHG) mainly responsible for global warming due to the burning of fossil fuels (coal, hydrocarbons, peat) for energy [1–3]. Other significant sources of atmospheric CO_2_ include byproducts from the fermentation of sugars, the respiration of all living organisms, volcanoes, hot springs, and geysers [4–7]. It is desired to develop green and sustainable technology to convert CO_2_ into valuable materials for carbon capture and sequestration (CCS) technology and reduce the burden on greenhouse gas emissions. Currently, the developed routes for CO_2_ reduction are the reduction at the source (i.e. reduced use of fossil fuels), sequestration and the chemical conversion [7]. Instead CO_2_ upcycling offers a pathway towards more sustainable processes [5, 8–10]. The conversion of CO_2_ into fuels or value-added chemicals is currently a field of great research interest. Non-enzymatic electrochemical and photocatalytic methods in recent years have been investigated for nicotinamide adenine dinucleotide (NADH) regeneration [11]. However, those non-enzymatic methods require a rare metal-chelating electron mediator or toxic methylviologen as prerequisites for the NADH regeneration routes [11]. Thus, in view of increasing environmental and sustainability considerations, seeking a green, sustainable, economic, and efficient regeneration method for NADH is indispensable.

**Fig 1:**
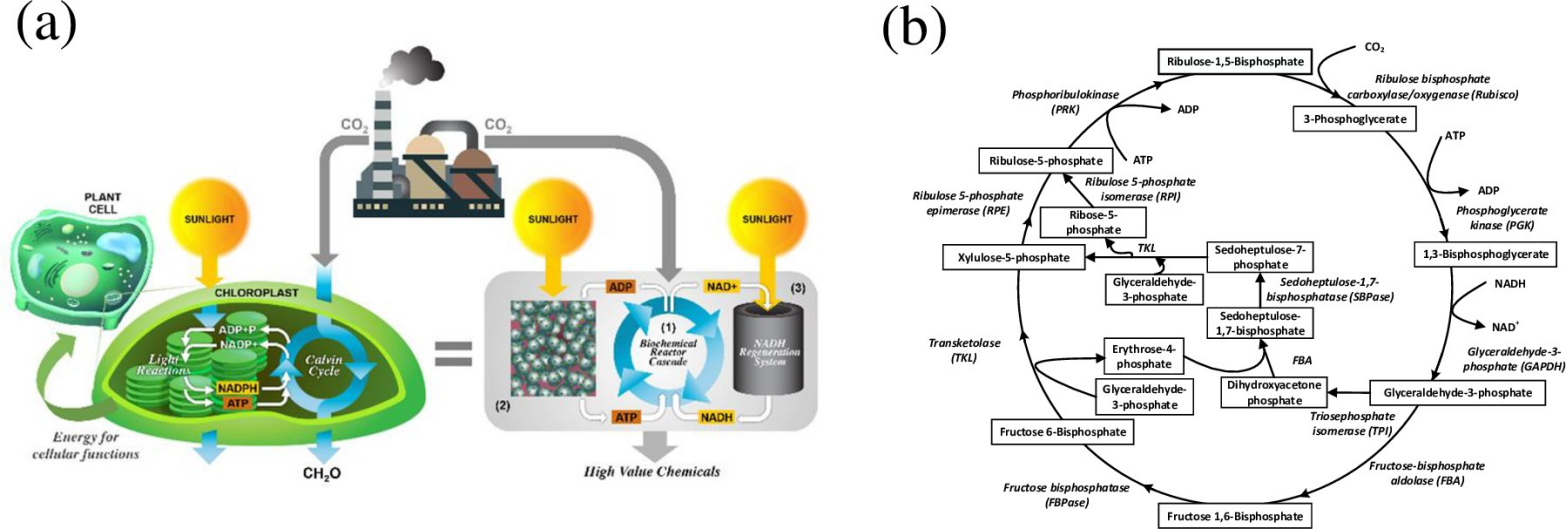
(a) A schematic of artificial photosynthesis mirroring plant cell photosynthesis: (1) Biochemical reactor cascade, (2) ATP regeneration system, (3) NADH regeneration system and (b) Calvin Cycle.

An alternate route to reconstruct cellular processes by imitating natural systems through photosynthesis is the Calvin cycle [12]. Herein, plants use CO_2_ and sunlight in a process commonly known as “photosynthesis” to produce organic matter. We aim to mimic the same principles in a non-living system, which we term “artificial photosynthesis” shown Schematically in Fig 1A along with Calvin Cycle Fig 1B, the driving engine of photosynthesis and our process.

It involves several enzymes along with light energy-dependent reactions ATP and NADH to form value-added products. First, CO_2_ is fixed from inorganic form to organic 3-PGA (3-Phosphoglycerate) which in turn forms an intermediate product 1,3-Bisphosphoglycerate in presence of PGK (Phosphoglycerate Kinase) and ATP. This intermediate along with GAPDH (Glyceraldehyde-3-Phosphate Dehydrogenase) and NADH forms G3P (Glyceraldehyde 3-Phosphate). Finally, G3P and ATP are used to regenerate RuBP (Ribulose-1,5-bisphosphate) and to fix more CO_2_ in the system [13–17]. In enzyme-catalyzed reactions, nicotinamide adenine dinucleotide (NAD^+^/NADH) redox couple, is an important component to synthesize precious medicinal and other value-added products [18–22]. The main function of cofactor NAD(H) is to supply electrons and hydrogen (proton) in these enzymatic reactions [11]. The 1,4-NADH is the only enzymatically active isomers among different reduced NADH. 1,4-NADH is rarely available isomer due to its high cost (ca. $992,000/kg). Therefore, it is very important to regenerate NADH *in-situ* and cost-effectively in a biochemical process for reuse. This will substantially help in the final product cost reduction and would, therefore, justify the use of such an expensive coenzyme 1,4-NADH.

Many studies have been reported in the literature on the synthetic Calvin Cycle [12, 23–27] to produce high-value chemicals also commonly called the *Reductive pentose phosphate pathway* [28]. It is an enzymatic cycle that catalyzes the photosynthetic assimilation of CO_2_ and produces pentoses. Ribulose bisphosphate carboxylase/oxygenase (RubisCO), the only enzyme capable of CO_2_ assimilation [28], is the first enzyme in the cycle and catalyzes the fixation of atmospheric CO_2_ to Ribulose 1,5-bisphosphate (RuBP). By CO_2_ fixation to RuBP two molecules of the triose phosphate, 3-Phosphoglycerate (3PGA), are produced. Through a series of nine enzymes, five of every six triose phosphate molecules produced by the Calvin Cycle are used for the regeneration of three molecules of RuBP. One triose phosphate molecule is the net gain from the fixation of three molecules of CO_2_, which a cell can use in a variety of biosynthetic processes.

By employing RubisCO’s ability to sequester atmospheric CO_2_ and the Calvin Cycle’s ability to regenerate the CO_2_ acceptor, the bioprocess developed in the current work can convert CO_2_ emissions into valuable small molecules without*-*bio or petro-based feedstocks. For example, through the application of various, well-known, enzymatic pathways the net gain of triose phosphate can be easily converted into hexose sugars such as fructose or glucose [28], glycerol, and numerous high-value chemicals including 2,3-butanediol, propionate, ethanol and butanol [26]. All of the necessary metabolic pathways share Dihydroxyacetone phosphate (DHAP) upstream of the final product formation. Nonetheless DHAP is the intermediate directly following 3PGA in the Calvin Cycle. By capitalizing on these metabolic pathways, we can easily tailor biochemical transformation pathways to convert 3PGA into higher-value chemicals.

The reducing power required by Calvin Cycle is supplied in the form of the enzymatic co-factor NADH. However, the regeneration system for a stable NADH has not yet been reported which is one of the key elements in artificial photosynthesis process system in order to convert CO_2_ into valuable products which are supported by co-factors ATP and NADH.

Several methods have been employed to reduce NAD^+^ to NADH [11, 29]. However, electrochemistry-based methods are very promising and have gained much attention for the regeneration of NADH. As widespread use of these methods is due to the intrinsic nature of electrochemistry when the reactants are electrons, the progress of the reaction can easily be monitored and controlled which can lead to relatively easy scale-up of the process [11, 30–33].

NADH regeneration via electrochemical reduction of NAD^+^ can be represented by the following established pathway [34]:

$$Step\;1:\;{\mathrm{NAD}}^{+}+e^{‐}\to \mathrm{NAD}•$$ (1)

$$Step\;2a\;\mathrm{NAD}•+e^{‐}+H^{+}\to \mathrm{NADH}$$ (2)

Step 2a (Eq 2) is the slow step where as a result enzymatically-inactive dimer NAD_2_ is produced due to the fast NAD-radical dimerization, step 2b [31, 35–40]:

$$Step\;2b:\;\mathrm{NAD}•+\mathrm{NAD}•\to {\mathrm{NAD}}_{2}$$ (3)

The high concentration of H^+^ will boost the reaction kinetics of Step 2a (Eq 2), as H^+^ will readily react with adsorbed NAD-radical on the electrode surface followed by Eley–Rideal mechanism, or Langmuir–Hinshelwood mechanism. When it follows the former mechanism, it will first adsorb on the electrode’s surface as M-H_ads_ and then react with the immediately available NAD-radical [34, 35, 41, 42]. In case, reaction follows the latter mechanism e.g. Langmuir–Hinshelwood, it results in the faster kinetics of Step 2a (Eq 2). In this case, the adsorbed hydrogen (H_ads_) on the electrode surface significantly affects recovery of the enzymatically active 1,4- NADH compared to inactive-dimer NAD_2_. However, it has been found that the concentration of adsorbed hydrogen, H_ads_ is strongly dependent on the electrode potential [34, 35, 41]. Therefore, hydrogen coverage or H_ads_ on the surface is an important parameter in the reduction of NAD^+^ to active NADH. Namely, on a bare glassy carbon (GC) electrode’s surface used in the developed system, recovery of enzymatically-active 1,4-NADH reached 98% at electrode potential of –2.3 V_MSE_ in the previous work, it was stated that highest NADH recovery can be obtained at this electrode potential [34, 35, 41]. The highest recovery was due to higher concentration of adsorbed ‘active’ hydrogen, H_ads_, at the more cathodic potential on the glassy carbon electrode surface.

In the current work, NAD^+^ was electrochemically reduced to enzymatically active 1,4-NADH which was subsequently oxidized to NAD^+^ in the simulated environment of Calvin cycle. The best experimental conditions were discussed and selected for electrochemical reduction of NAD^+^ to enzymatically active NADH to produce different sugars. The optimized experimental conditions for the electrochemical regeneration of NADH were presented and discussed in contest of literature. In the second part of the study, the integration of NADH regenerative system with ATP in a biochemical reactor containing all the essential ingredients for Calvin cycle were presented. To the best of authors’ knowledge, no published literature is available on the regeneration of NADH through electrochemical method during Calvin Cycle (artificial photosynthesis) and is, therefore, the focus of the current work.

The synthetic Calvin Cycle engineered by Ingenuity Lab (Fig 1A and 1B) has the potential to produce greater than fifty high-value chemicals. However, it is very important to develop a simple and stable NADH regeneration system which is one of the key elements in artificial photosynthesis process system since the energy required for the conversion of C O_2_ into organic molecules is supported by co-factor molecules ATP and NADH.

The technology is a multi-enzyme platform that generates valuable, small organic molecules from CO_2_ produced by industrial processes, sunlight, water, and electricity. It essentially provides photosynthesis without the energy requirements for reproduction and growth found in traditional biological carbon fixation platforms. The process is designed as a cascade of bioreactors, which allows for optimized reaction conditions at each stage of the process.

## Materials and methods

### Chemicals and solutions

Enzymatically active NADH regeneration was performed in 50 mM Tris-buffer solution containing 0.2 mM of NAD^+^ (purity 95%, Sigma N0632) in a conventional three-electrode electrochemical batch reactor. The initial volume of the electrolyte in the reactor was 80 mL at a constant pH of 7.4 and 295 K. Deionized water (resistivity 18.2 MW cm, Milli-Q® systems) was used in the preparation of Tris-buffer solution which is used to adjust of pH of Hydrochloric acid. No further purification of the chemicals was carried out and were used as received.

### Electrochemical cell and electrodes

**Fig 2:**
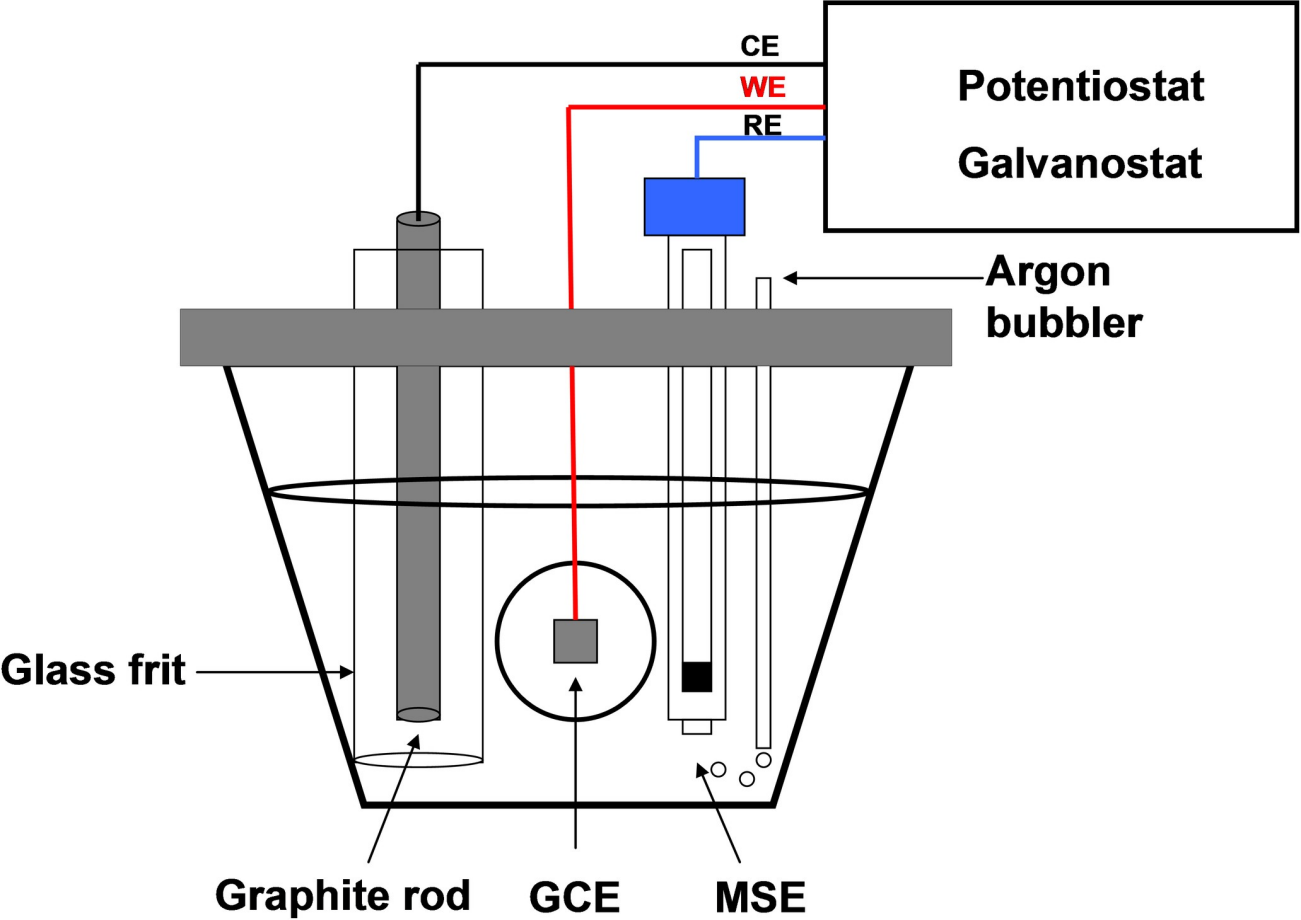
Three-electrode batch electrochemical reactor (cell) used in the research.

A conventional three-electrode based electrochemical batch reactor/cell was used as shown in Fig 2.

Glassy carbon (GC) electrode with two different geometric areas (12.5 and 50 cm^2^) was used as a working electrode (cathode) in the reactor. Graphite rod from McMaster-Carr 9121K71 was utilized as an anode (counter electrode). Initially, the anode was sonicated for 30 min in ethanol followed by rinsing with deionized water. Since oxygen evolves on the anode during the NADH regeneration, therefore it was separated from the bulk electrolyte using glass tubes with Nafion membrane to prevent the diffusion of dissolved oxygen to the working electrode. As a reference electrode, a Mercury/mercurous sulphate electrode (MSE; +0.642 V vs. SHE) supplied by Fisher Scientific was used and all the reported potentials in this paper are with reference to MSE. The experiments were carried out under the optimized conditions, for example, at electrode potential of -2.30 V vs. MSE, room temperature, and biological pH. Detailed experimental procedure and essays have been described in S1 File.

### Equipment

Linear polarization, cyclic voltammetry, and controlled-potential electrolysis techniques were carried out by utilizing Ecochemie Autolab potentiostat/galvanostat PGSTAT30/ controlled using the NOVA v.12.1.1 software. An in-line OceanView UV-Vis spectrophotometer was utilized to track the reduction kinetics of NAD^+^ (NADH regeneration). The activity assay was monitored by a Perkin Elymer Lambda 1050 UV-Vis-Nir Spectrophotometer at a wavelength of 340 nm. The detailed procedure of the assay can be found in the literature [31, 35, 36, 41, 43].

### Electrode pretreatment

**Fig 3:**
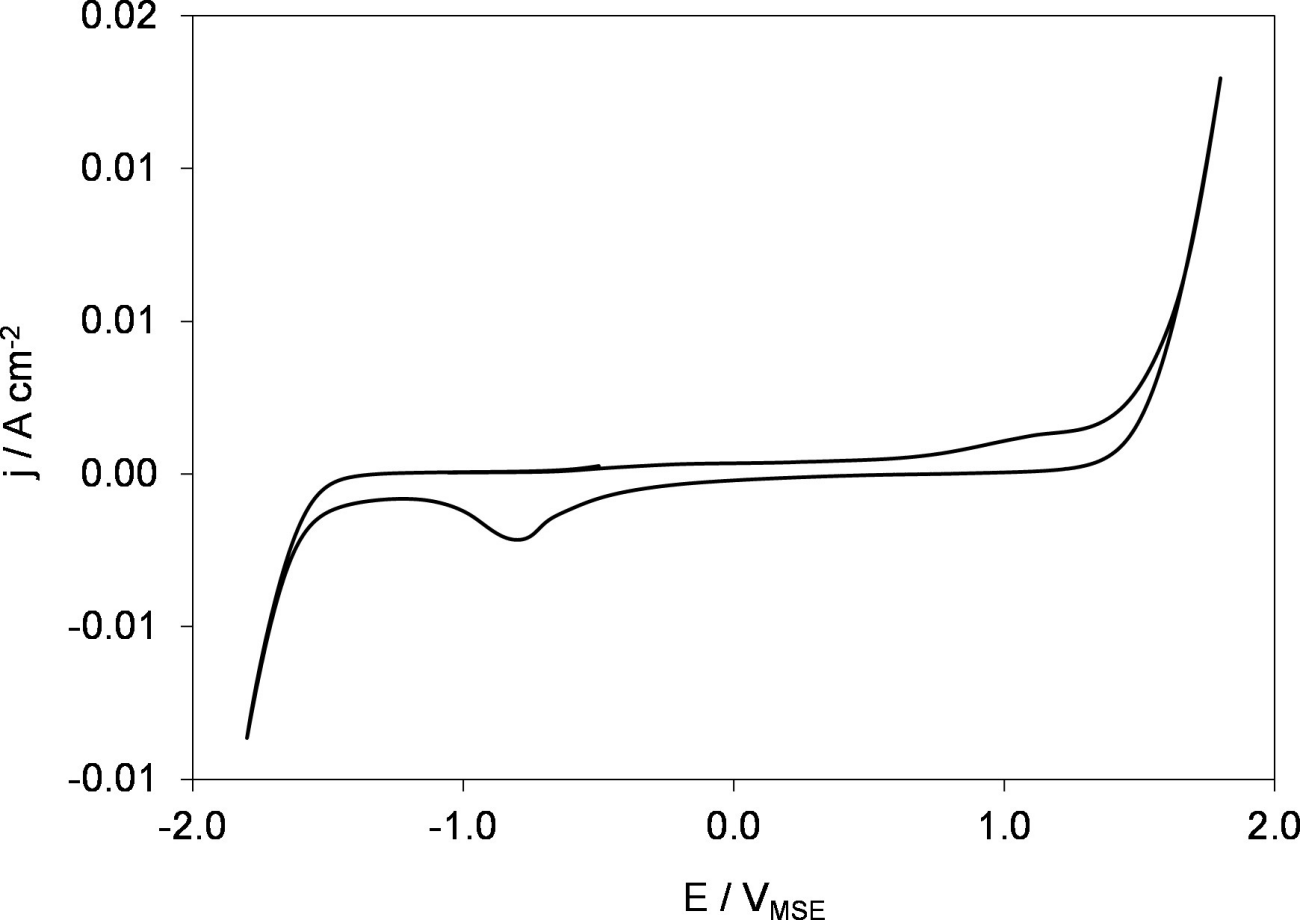
Cyclic voltammogram of GC electrode in 0.5 M H_2_SO_4_ solution. Scan rate, *sr*: 100 mV s^–1^. Temperature, *T = 295 K*.

Before the NADH regeneration experiment, a pretreatment wet-polishing of the GC surface was done with 1200/4000 grid paper to achieve a mirror-finished surface. It was degreased with ethanol followed by sonication in ethanol for 30 min to get rid of any residues from the electrode surface during electrode pretreatment. Since impurities on the electrode surface greatly affect its performance, GC electrode was electrochemically cleaned in 0.5 M H_2_SO_4_ (Fisher Scientific 351293) by cyclic voltammetry between –1.8 and 1.8 V at a scan rate of 100 mV s^–1^, for 50 cycles using the same electrochemical reactor. A very stable and reproducible cyclic voltammetry (CV) profile confirmed that the surface of the GC electrode is very clean as shown later in Fig 3.

### Electrochemical regeneration of active NADH

Electrochemical regeneration of active NADH was performed in the electrochemical reactor (Fig 1). As counter electrodes, two graphite rods were used, and GC electrode was used as a working electrode in the developed batch electrochemical reactor. For a uniform electric field in the reactor, counter electrodes were arranged and placed opposite to both surfaces of working electrode. A total electrolytic volume of 80 mL was used with an initial 0.2 mM concentration of NAD^+^.

### Experimental methodology

All measurements were performed in an oxygen-free electrolyte. To achieve this, pure argon (99.9%) was purged through the electrolyte for 30 min prior to and during electrochemical NADH regeneration. This also ensured convective mass transport of electroactive species to/from the electrode surface since NAD^+^ reduction reaction is mass-transport controlled reaction.

To investigate the enzymatic activity of the regenerated NADH, activity tests were made according to the regular Sigma Quality Control Test Procedure (EC 1.8.1.4) which was further modified for this purpose using lipoamide dehydrogenase (5.3 U/mg, Calzyme laboratories, Inc. 153A0025) as an enzyme and DL-6,8-thioctic acid amide (MedChem Express, HY-B1142) as a substrate [36, 44].

The Calvin Cycle started with sequestering CO_2_ in the RuBisCO foam reactor. While CO_2_ sparged through the liquid, it formed the foam architecture due to the presence of ranaspumin-2 (RSN-2) as a surfactant. The enzyme solution was recycled from the bulk of the liquid in the bottom of the reactor to the top, flowing through the foam structure. This allows for CO_2_ to come into contact and react with RuBP in the presence of RuBisCO to produce 3PGA, an intermediate in the Calvin Cycle and the feed for the next reactor. 3PGA was separated through an ultrafiltration (UF) system and proceeded to the next reactor. Except for the RuBisCO reactor, in which the enzyme was in the bulk of the solution, i.e. dissolved free form, all other reactors used enzymes that are immobilized onto beads and packed into the reactor space. The reactants passed through a packed bed of immobilized enzyme where the reactions took place. The reactors were designed such that the reactants were completely converted to the product as they passed along the packed bed. Immobilization eliminates the need to separate the enzyme from the solution before going to the next reactor. The reactors that involve cofactors (ATP or NADH) have nanofiltration (NF) separation system to separate the cofactors to be regenerated and recycled. When possible, other reaction steps were combined into multi-bed reactors, where immobilized enzymes were sequentially packed, and reactants while passing along the beds were converted to the reactants of the subsequent bed. For details, S1 File may be read. Experimental work was repeated at least three times and the mean values was considered. The standard error among the triplicate readings was withing ±5%.

## Results and discussion

### Activation of glassy carbon electrode

To minimize the interference of hydrogen evolution reaction (HER) and its effect on NAD^+^ reduction reaction, Glassy Carbon was used as a working electrode (cathode) due to its high hydrogen reduction overpotential, in addition to low porosity and comparatively better electrical conductivity [45]. Furthermore, it is cheap, easily available, and stable under extreme experimental conditions, which make it suitable amongst other materials for industrial applications [45]. During electrochemical measurements, surface of the electrode material plays an important role. Cleanliness from impurities and surface chemistry of carbon-oxygen functionalities of the electrodes greatly affect these measurements [46]. Therefore, GC electrode was electrochemically cleaned in 0.5 M H_2_SO_4_ (Fisher Scientific 351293) by cyclic voltammetry between –1.8 and +1.8 V at a scan rate of 100 mV s^–1^, for 50 cycles using the same electrochemical reactor. The results are plotted in Fig 3.

Fig 3 demonstrates a very stable and reproducible cyclic voltammogram (CV) which clearly indicates a clean GC electrode. Namely, it is characterized by a large double layer (DL) region of glassy carbon electrode negative of +1.0 V and positive of –1.0 V [47, 48]. There is no evidence of any redox reaction occurring on the GC surface since the current (reaction rate) is zero. The increase in the current positive of +1.0 V is indication of oxygen evolution reaction (OER) while the increase in the current negative of –1.0 V due to the HER. Thus, the electrochemical activation of GC involves both oxidation and reduction of the GC electrode surface.

### Linear polarization voltammetry (LV)

**Fig 4:**
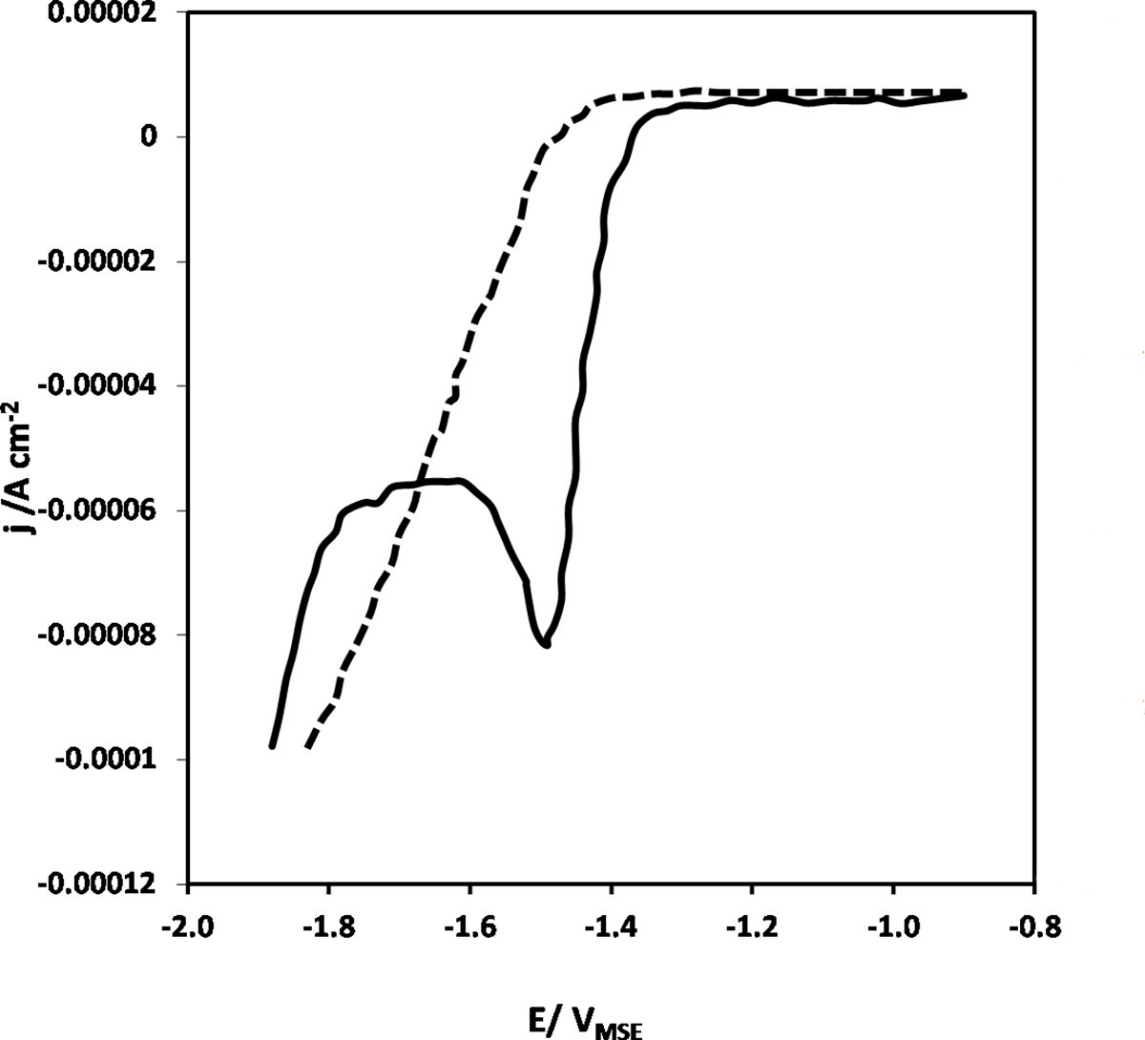
Linear voltammogram of GC electrode in 0.1 M phosphate buffer solution (dashed line) and phosphate buffer solution containing 4 mM NAD^+^ solution (solid line) at a scan rate (*sr) of* 10 mV s^–1^. Temperature, *T = 295 K*.

Linear voltammetry (LV) was conducted in order to determine the working potential region of NAD^+^ reduction to active NADH as shown in Fig 4.

Initially, a linear voltammogram was recorded only in the buffer solution without NAD^+^ molecule (dashed line) which shows a control curve of the glassy carbon electrode that is atypical behavior of the pure GC electrodes under the applied experimental conditions [35]. Such behavior of GC electrode is attributed to a wide double layer region (positive of ca. –1.40 V) and the beginning of HER (negative of ca. –1.50 V). On the other hand, when the same experiment was carried out in the electrolyte containing 4 mM NAD^+^ (solid line), one can see a broad and sharp cathodic current peak at a potential negative of ca. –1.30 V as shown in Fig 4.

Two major potential regions can be identified in Fig 4 (solid line), i.e. the region negative of ca. –1.30 V is related to the NAD^+^ reduction region with a well-pronounced NAD^+^ reduction peak negative of ca. –1.40 V. This cathodic current peak is of NAD^+^ reduction reaction on different electrode materials as evident from literature [31, 37, 49–53]. Thus, LV technique resulted in some useful information on NAD^+^ reduction reaction. However, in the actual biochemical reactor, 1,4-NADH regeneration would not be performed under potentiodynamic conditions, but rather potentiostatic. For this purpose, controlled-potentiostatic measurements were carried out in order to reduce NAD^+^ to active NADH.

### Electrochemical regeneration of active NADH

Electrochemical NADH regeneration was studied in various buffer solutions at different concentrations and temperatures in order to optimize the experimental conditions for faster reaction kinetics and high yield of NADH.

### Phosphate buffer solution

**Fig 5:**
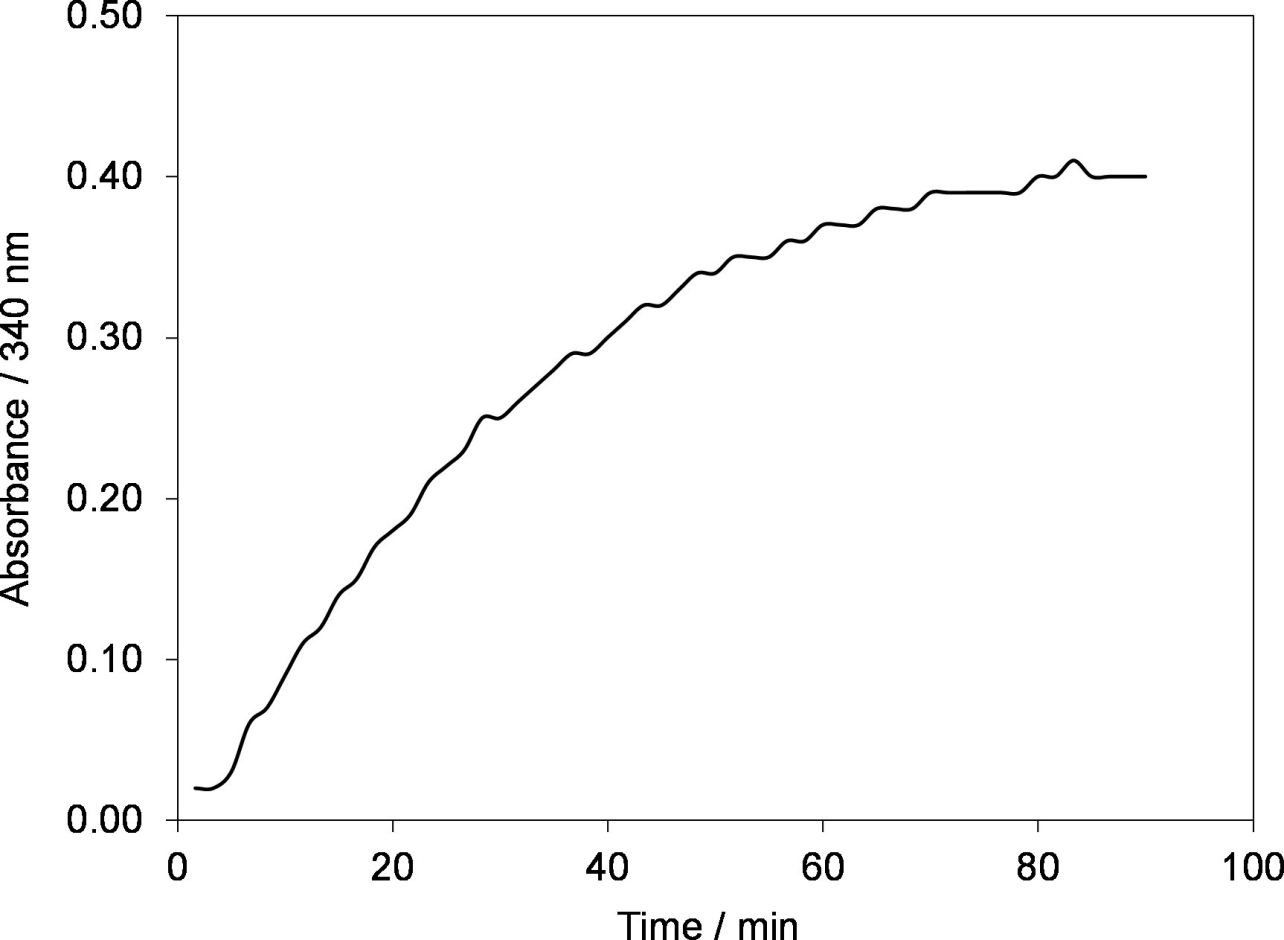
Time evolution of absorbance at 340 nm during electrolysis of 0.2 mM NAD^+^ in phosphate buffer solution at electrode potential of –2.30 V using GC electrode (geometric area, A = 50 cm^2^). Temperature, *T = 295±2 K*.

Initially, the NAD^+^ reduction kinetics and recovery of regenerated active 1,4-NADH were investigated in Phosphate Buffer solution at room temperature (295 K ±2) and pH 7.4 at selected electrode potential of –2.30 V as shown in Fig 5.

Fig 5 shows the time dependence of absorbance at 340 nm at electrode potential of –2.30 V using Glassy carbon electrode. In the beginning, the absorbance at 340 nm is zero. However, when electrode potential of –2.30 V was applied, the absorbance started to increase with time. After 70 min of electrolysis the absorbance reached a plateau indicating the completion of the reaction. As explained earlier in the manuscript that at 340 nm both NAD_2_ and 1,4-NADH absorb, hence Fig 5 does not give the precise information about active NADH regenerated rather it only shows the conversion of NAD^+^ to both 1,4-NADH and NAD_2_ the latter being enzymatically-inactive [31, 35, 36, 41, 43]. Therefore, to distinguish between the two species, an enzymatic assay was conducted. Briefly, the assay uses DL-lipoamide as a substrate to convert it into dihydrolipoamide in the presence of lipoamide dehydrogenase as enzyme, represented by Eq (4) [44]

$$1,4-\mathrm{NADH}+\mathrm{DL}‐lipoamide\overset{lipoamide\;dehydrogenase}{\to }NAD^{+}+dihydrolipoamide$$ (4)

Oxidation of NADH to NAD^+^ reduces absorbance at 340 nm, which clearly can be seen and observed. In order to calculate the percentage of active NADH produced, the absorbance at 340 nm before and after reaction (4) was utilized. It is very important to mention here again that both active NADH (and its different isomers) and NAD_2_ absorb at 340 nm and thus cannot be used to determine the actual amount of active NADH. For this purpose, an activity assay was conducted for the samples collected from the reactor. The results of the activity assay show that 97.45±0.8% of active 1, 4-NADH was recovered under the experimental conditions employed which is in agreement with our previous studies [34, 35, 41]. Now, taking that the NAD_2_ formation requires one electron per one NAD^+^ molecules, while the formation of 1,4-NADH requires the exchange of two electrons (Eq 2), the 97.45% active NADH indicates that 1.97 electron went to reduce NAD^+^ to active NADH. The remaining could be both inactive NADH isomers or/and dimer NAD_2_. Thus, almost 98% of electrons went to reduce NAD^+^ to active NADH.

**Fig 6:**
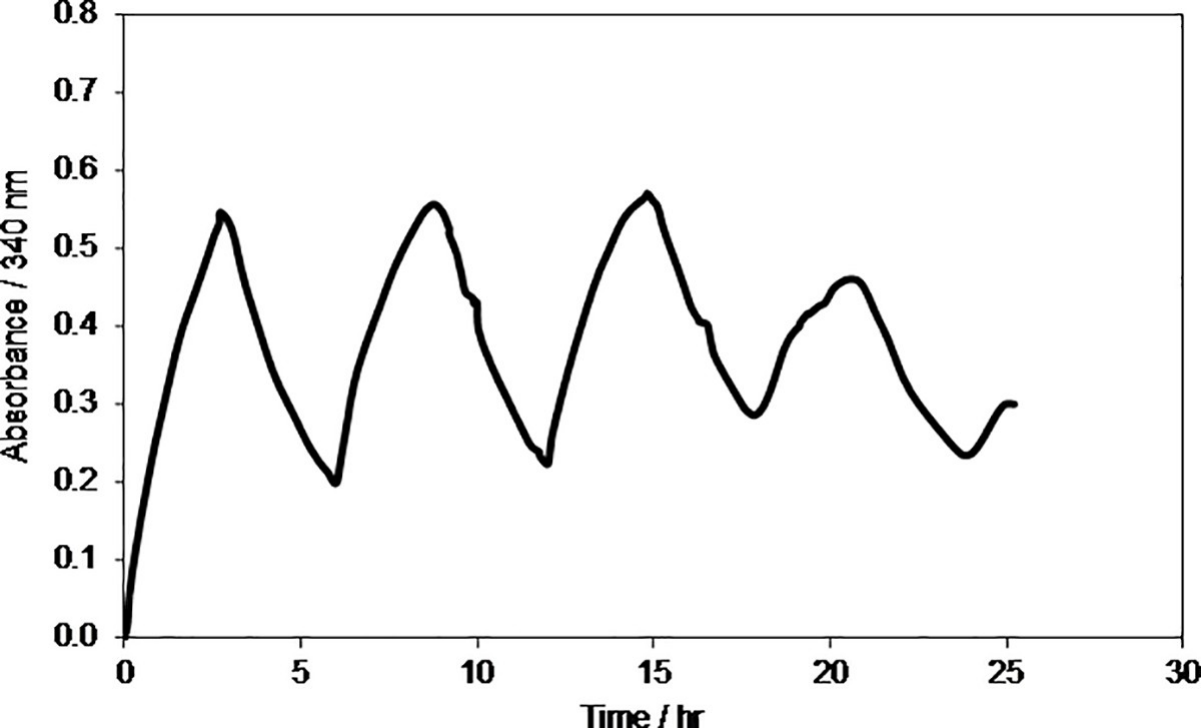
Time evolution of absorbance at 340 nm during electrolysis of 0.2 mM NAD^+^ in phosphate buffer solution during reduction and oxidation cycles at electrode potentials of –2.30 V and +0.40 V, respectively using GC electrode. Temperature, *T = 295±2 K*.

**Fig 7:**
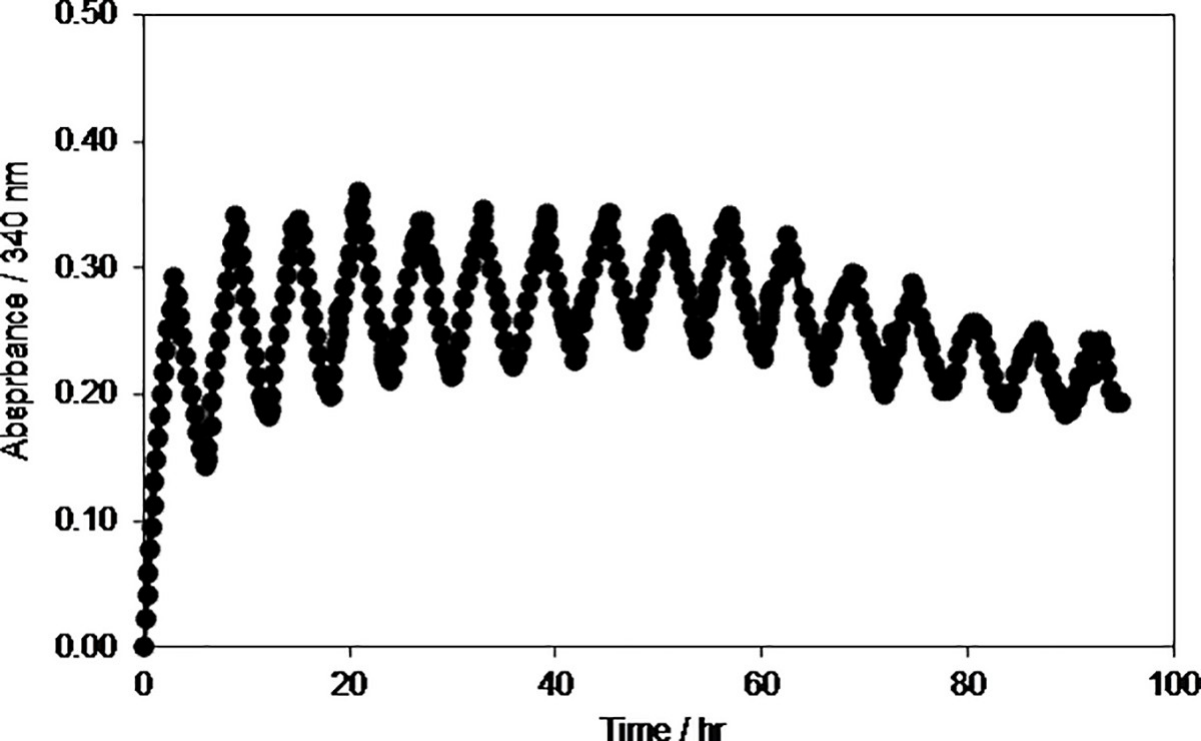
Time evolution of absorbance at 340 nm during electrolysis of 0.2 mM NAD^+^ in phosphate buffer solution during reduction and oxidation cycles at electrode potentials of –2.30 V and +0.40 V, respectively using GC electrode. Temperature, *T = 277±2 K*.

To investigate if regenerated 1,4-NADH is stable for a prolonged time and can be oxidized back to NAD^+^ electrochemically, cyclic reduction and oxidation were carried out at two different temperatures i.e. at room temperature, *T = 295±2 K* and low temperature, *T = 277±2 K* as shown in Figs 6 and 7, respectively.

The results in Fig 6 demonstrate that NAD^+^ indeed was reduced to NADH as the absorbance at 340 nm increased with time from 0 to 0.55 at –2.30 V using GC electrode and it is reached to its maximum value in approximately 3 hrs that shows the completion of NAD^+^ reduction reaction [41]. The GC electrode of small geometric area (12.5 cm^2^) was used as compared to 50 cm^2^ (Fig 5) which took only 70 min. Soon after the completion of reduction step, the oxidation of the regenerated NADH was initiated back to NAD^+^ at electrode potential of +0.40 V using the same GC electrode. As it can be seen in Fig 6 that the absorbance at 340 nm started to decrease showing the oxidation of NADH to NAD^+^ since NAD^+^ absorbs at 260 nm. The cycle was repeated in order to investigate how many cycles the system can regenerate NADH? It was noticed that with each reduction cycle the absorbance at 340 nm decreased and after 30 hr, the developed system was not able to reduce or oxidize the NAD(H) molecule. Thus, it was concluded that at room temperature the NAD^+^/NADH is stable for a maximum period of 24 to 30 h (4 cycles).

Further, to investigate the effect of temperature on the oxidation/reduction cycle, the experiments were repeated under the same conditions but at a low temperature of 277 K as shown in Fig 7.

It was very interesting to see that at low temperatures the system was able to continuously reduce and oxidize the NAD^+^/NADH up to 96 hrs (Fig 7) as compared to 30 hrs at room temperature (Fig 6). Thus, it could be concluded that at low temperature the NAD(H) is stable for 96 hrs under the applied experimental conditions. These results show that the regenerated NADH was active for 96 hrs. Thus, the formed radicals do not affect the chemical structure of NADH and enzyme under the applied experimental conditions.

### Tris-buffer solution

**Fig 8:**
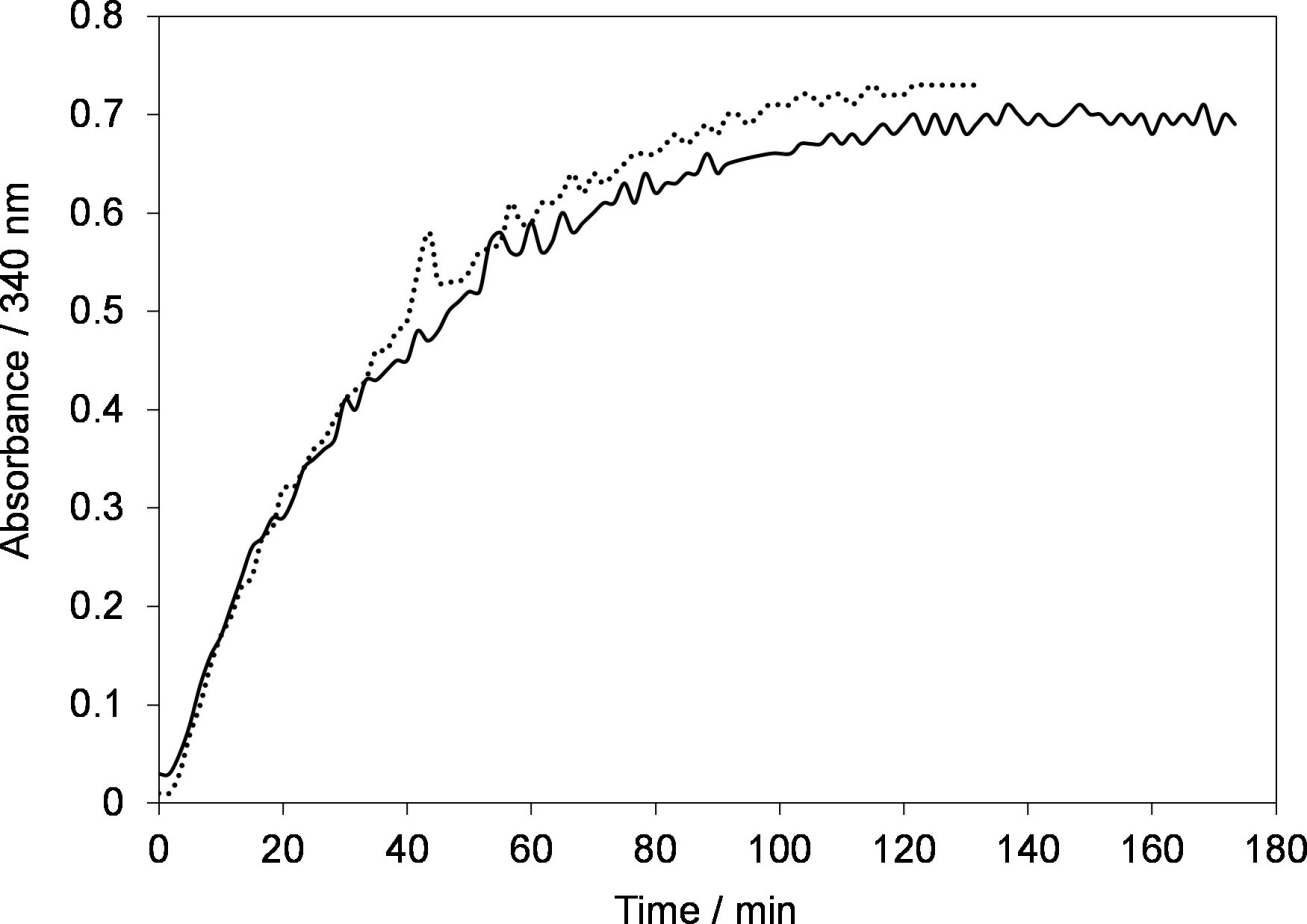
**Time evolution of absorbance at 340 nm during electrolysis of 0.2 mM NAD**^**+**^ **in 100 mM Tris-buffer solution (dotted line) and 260 mM (solid line) at electrode potential of –2.30 V using GC electrode.** Temperature, *T = 295±2 K*.

Further to study the effect of buffer solution on the kinetics of NAD^+^ reduction reaction and recovery of active 1,4-NADH, experiments were conducted under the same operating conditions (Fig 5) but in Tris-buffer with two different concentrations 100 (dotted line) and 260 mM (solid line) as shown in Fig 8.

Fig 8 shows that the kinetics of NAD^+^ reduction reaction is a bit slower than in phosphate buffer (Fig 5) and it takes longer to reach a plateau. However, the conversion is much higher than the phosphate buffer solution. Namely, based on the standard curve for commercial NADH (figure not shown here) only 50% of initial NAD^+^ was converted to the products using phosphate buffer solution. However, the conversion increased to 70% when Tris-buffer was used. The possible reason for low conversion using the phosphate buffer solution could be the adsorption of phosphate groups on the electrode surface which blocks the electrode surface and thus, minimize the electrochemically active surface area (EASA) of the electrode. In addition, it is very clear from Fig 8 that the concentration of Tris-buffer has no significant effect on the regeneration kinetics of NAD^+^ reduction reaction.

Hence, it was found from these experiments that 100 mM Tris-buffer solution resulted an improved conversion of NAD^+^ to the reduction products compared to phosphate buffer solution. Furthermore, the kinetics of NAD^+^ reduction is slightly slower than phosphate buffer solution and the concentration of the Tris-buffer solution has no effect on both reduction kinetics and recovery of active NADH.

**Fig 9:**
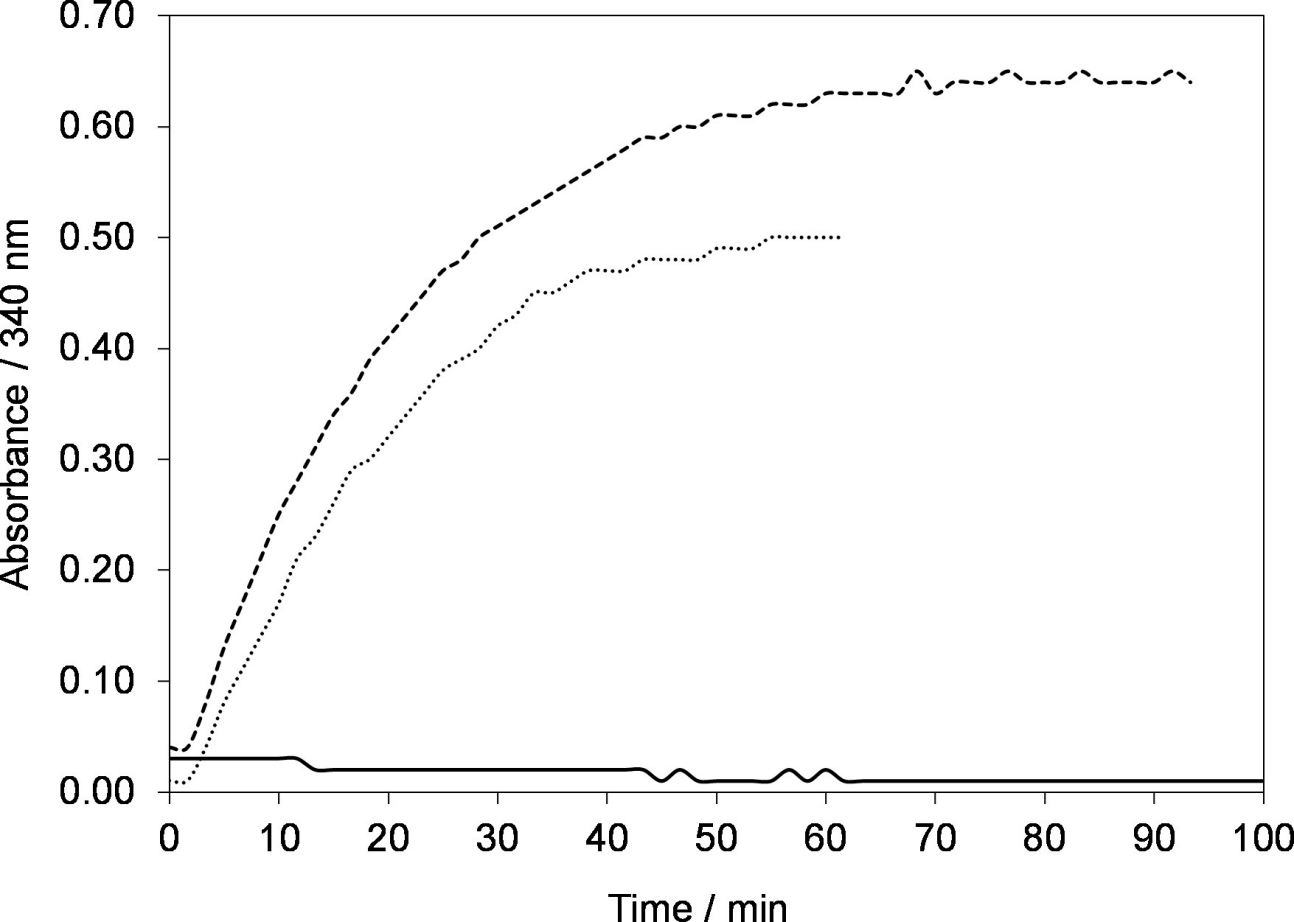
**Time evolution of absorbance at 340 nm during electrolysis of 100 mM Tris solution containing 0.2 mM NAD**^**+**^ **and 10 mM MgCl**_**2**_ **(dotted line) and 0.2 mM NAD**^**+**^**, 10 mM MgCl**_**2**_ **and1 mM DTT (dashed line) at electrode potential of –2.30 V using GC electrode and control experiment (solid line), no potential applied.** Temperature, *T = 295±2 K*.

In order to replicate the Calvin Cycle in a real biochemical reactor, other chemicals and enzymes will also present in the reaction mixture. Thus, we performed the NADH regeneration experiment with a reaction mixture of 10 mM MgCl_2_, 1mM Dithiothreitol (DTT) and 0.2 mM NAD^+^ in selected 100 mM Tris-buffer at pH 7.4 and room temperature as shown in Fig 9.

The trend in Fig 9 (dashed line and dotted lines) exhibits similar regeneration kinetics. However, the conversion is decreased from 70 to 50% which may be due the adsorption of MgCl_2_ on the surface of electrode. However, it was interesting to see that conversion increased from 50 to 60% again with the addition of DTT (dashed lines). Since DTT is reducing agent therefore to see the reducing effect of DTT on the NAD^+^ reduction, we performed a control experiment without applying potential Fig 9 (solid line). As the absorbance at 340 nm is zero throughout the time of the experiment, therefore it confirmed that the increase in regeneration kinetics was not due to the reducing effect of DTT.

**Fig 10:**
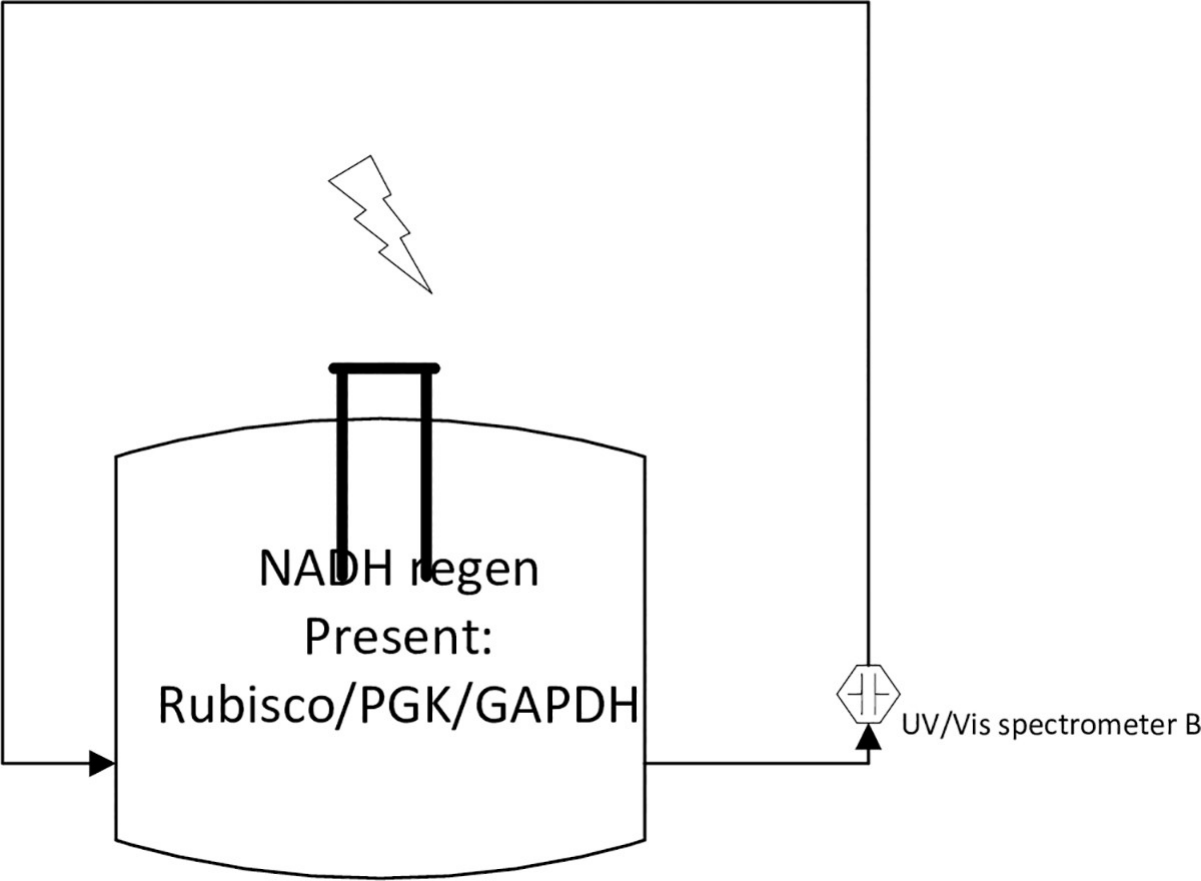
Schematic of NADH regeneration module using free enzymes.

**Fig 11:**
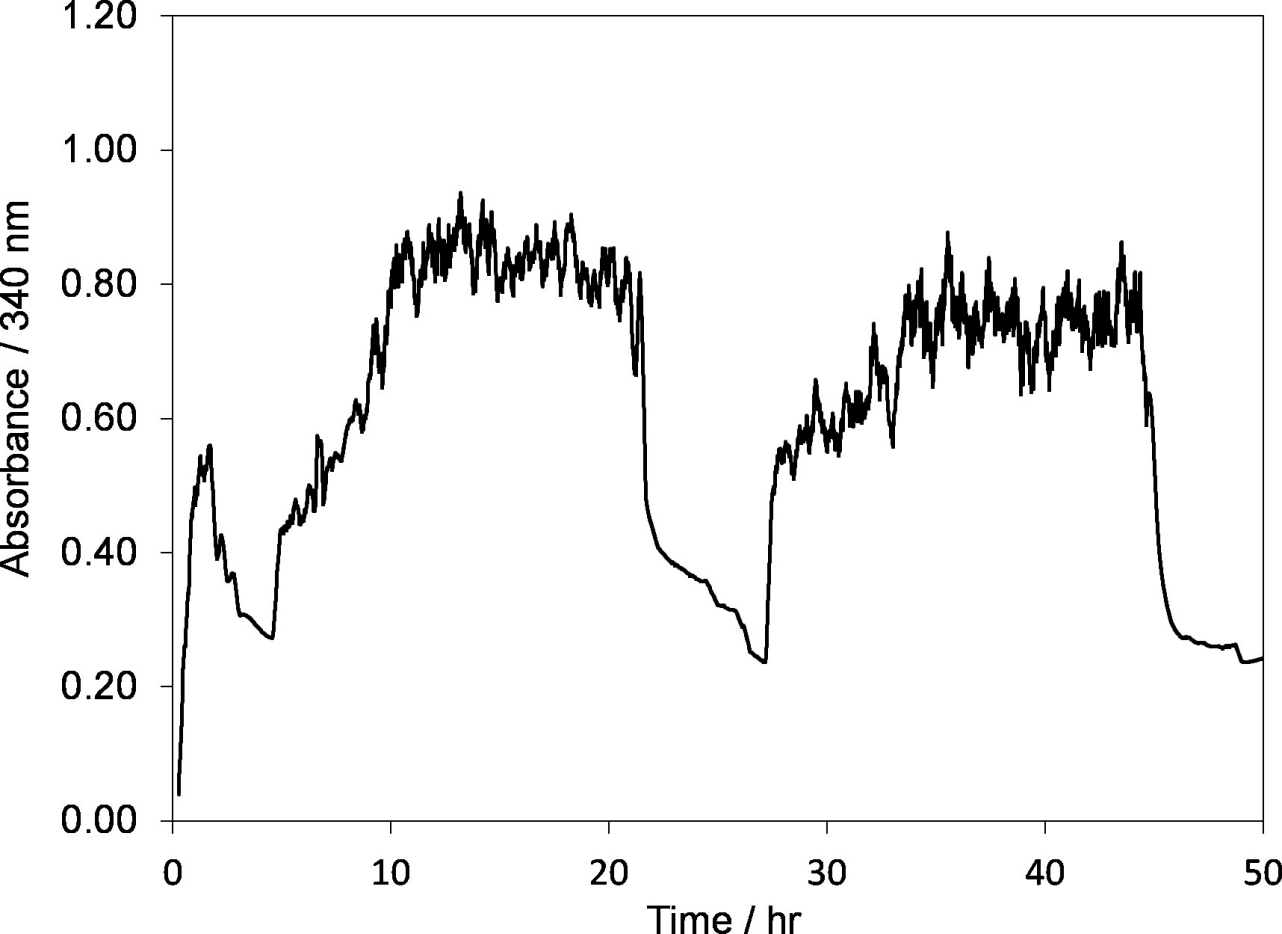
Time evolution of absorbance at 340 nm during free enzymes cycle containing 0.2 mM NAD^+^ at an electrode potential of –2.30 V using GC electrode. Temperature, *T = 295 K*.

The next step in the process was to integrate NADH regeneration system with other essential modules of the Calvin Cycle. However, before the NADH regeneration integration with other modules, we tested the system with free enzymes in order to make sure that the system is working. Therefore, experiments were conducted to study the regeneration of a system that contained free enzyme as well as NAD^+^ as shown in Fig 10 and the corresponding results are shown in Fig 11.

Fig 11 clearly shows that initially the absorbance at 340 nm is zero confirming that there was no NADH in the system. However, the absorbance at 340 nm increases with time when an electrode potential of –2.30 V was applied. Here, it is important to mention that both NADH and NAD_2_ absorb at 340 nm but as we already showed in the first part of the manuscript that our developed NADH regeneration system yields 97.45±0.8% of active NADH which is confirmed by the decrease in absorbance in Fig 11 since according to Eq 4, only enzymatically-active NADH is consumed during the reaction and not enzymatically-inactive NAD_2_. Therefore, the peak at 340 nm here corresponds to active NADH, not NAD_2_. Cyclic regeneration was performed and the NAD^+^ was reduced 3 times over the 45 hr test period.

### System integration

**Fig 12:**
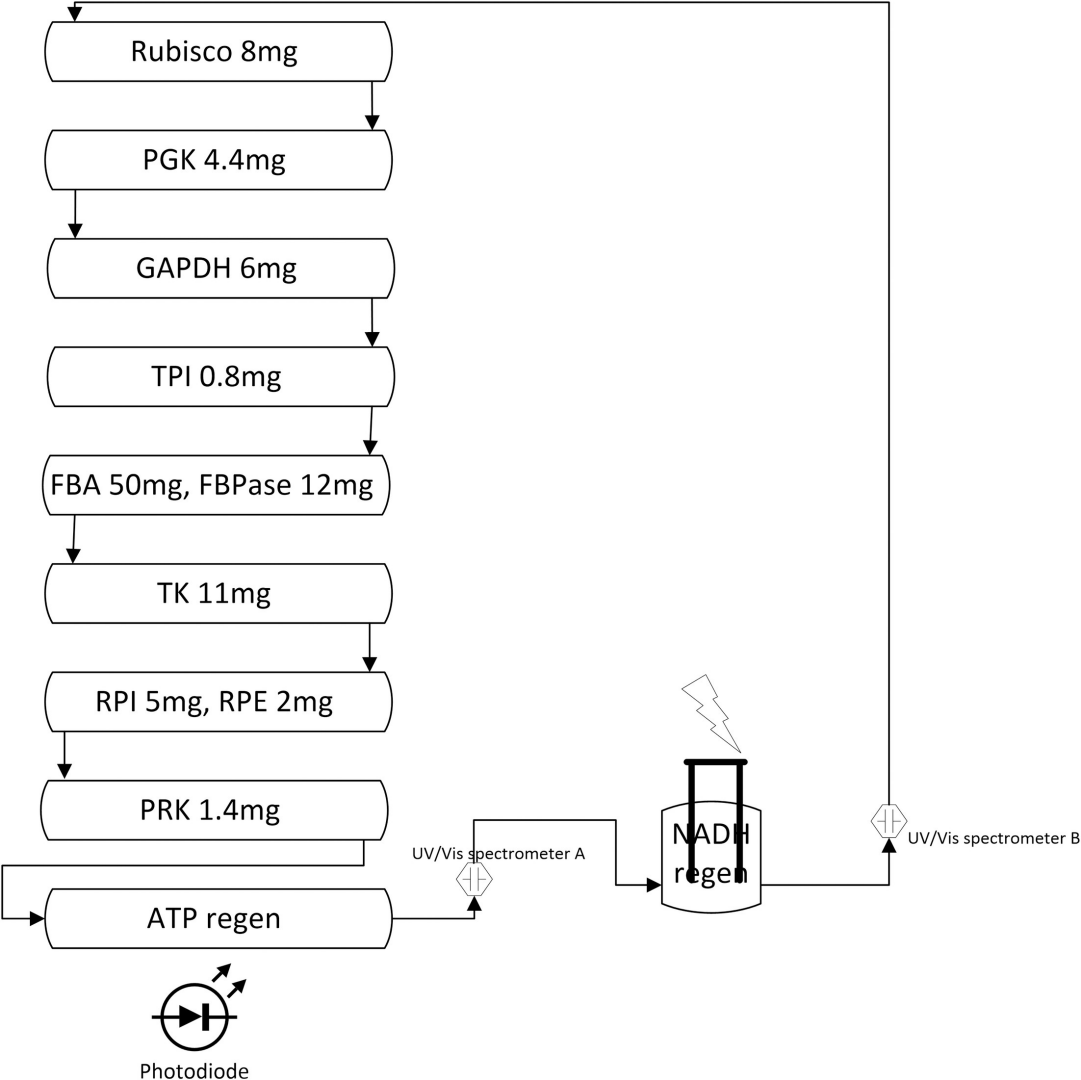
Schematic of the full Calvin cycle experiment including all three essential modules.

The key modules in artificial photosynthesis are: (i) adapted Calvin Cycle and biochemical transformation which include enzyme production, separation, purification, and immobilization, (ii) light-driven ATP synthesis and (iii) electrochemical regeneration of NADH. Fig 12 shows the schematic of corresponding integration of all modules into one system.

**Fig 13:**
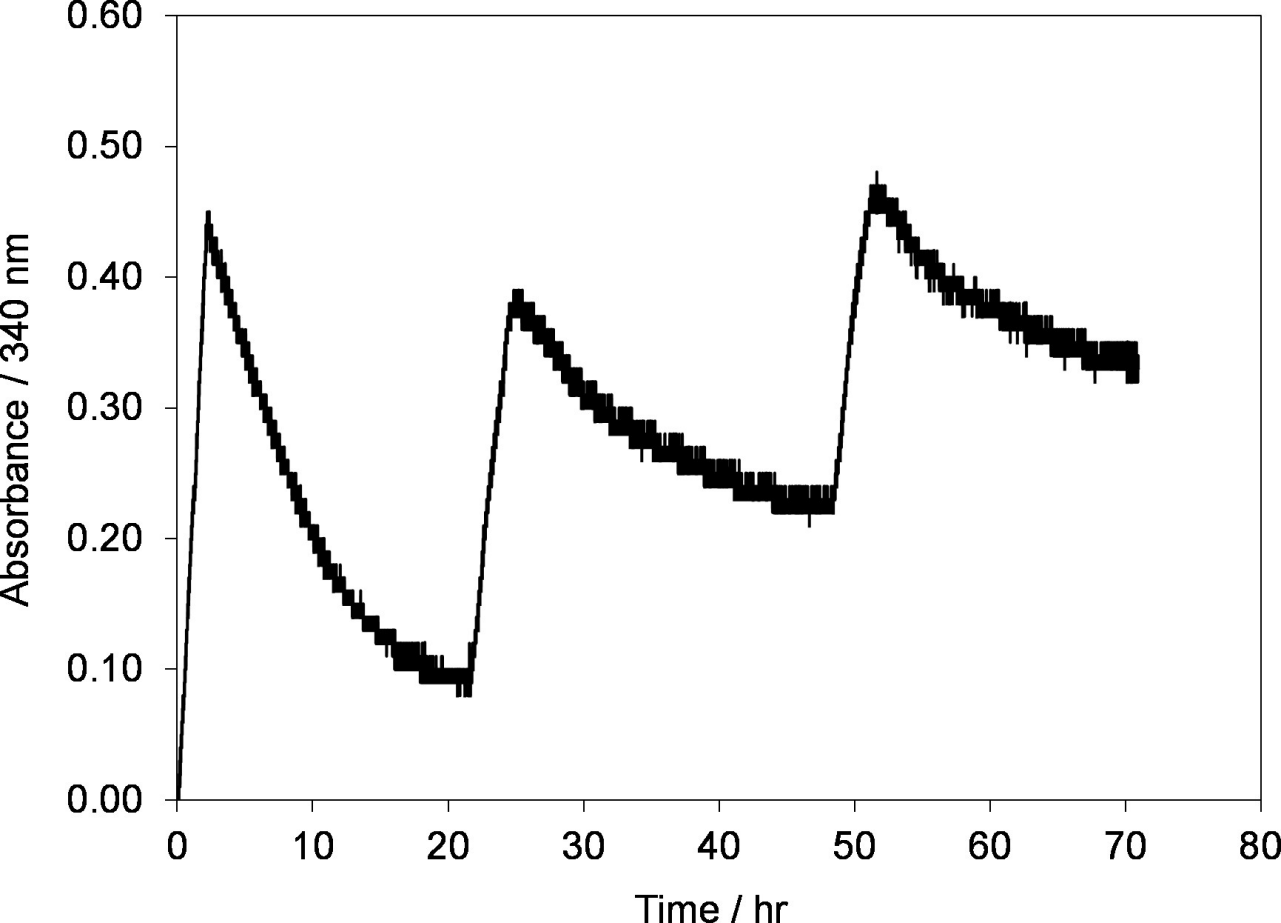
Time evolution of absorbance at 340 nm during full cycle experiment containing 0.2 mM NAD^+^ at an electrode potential of –2.30 V using GC electrode. Temperature, *T = 295±2 K*.

For the integration of all three modules the enzymes of the Calvin Cycle were immobilized in hollow fiber system. The corresponding enzymes and their amounts are shown in Fig 12. CO_2_ was provided in the form of bicarbonate $\left({HCO}_{3}^{-}\right)$. NAD^+^ was regenerated using a GC electrode in the same setup (Fig 2) at an applied potential of –2.30 V and ATP was generated by vesicles that were trapped and recirculated in the final hollow fiber system. To measure the NADH concentration based on absorbance at 340 nm, two UV/Vis spectrometers were used. The full cycle experiment was conducted over multiple days as shown in Fig 13.

As the system has no NADH at the start, therefore the absorbance at 340 nm is zero. However, when an electrode potential of –2.30 V was applied, one can observe a sharp increase in the absorption at 340 nm. Over the 3-day period, regeneration was done 3 times (3 cycles) which can be seen as sharp increases in Fig 13. The corresponding rates for NADH regeneration and disappearance during the 3 cycles are shown in Table 1. The concentration of regenerated ATP in the integrated system was around 35 μM.

**Table 1:** NADH regeneration and consumption rates.

Cycle (day)	1	2	3
Regeneration (μM min^-1^)	0.50	0.20	0.19
Disappearance (μM min^-1^)	0.04	0.02	0.02

ATP was generated through coupled activity of proton pumping by bR in presence of LED light to the vesicles trapped in the last hollow fiber system. ATP produced was measured by Luciferin-Luciferase assay kit. NAD^+^ was regenerated to NADH over 2 hr. Then, NAD^+^ regeneration was stopped to allow the detection of its reduction over a one-day span. This would allow for confirming of successful integration of NADH and ATP regeneration subsystems. Then, regeneration was done again to replenish NADH concentration. To the best of authors' knowledge, no such system has been reported in the literature till now.

The different modules involved in this technology including ATP regeneration and NADH regeneration were individually developed successfully. It was showed that we can integrate the system and run all the modules. However, the methodology for immobilization of the enzymes needs to be modified since it was not efficient as expected. Although, we could integrate the system successfully, the amount of capturing CO_2_ was really low due to the significant decrease in efficiency of the enzymes after immobilization. Therefore, we are currently working on other immobilization techniques with minimum loss of efficiency.

Please note that the data in Table 2 is only related to NADH regeneration from commercially available NAD^+^ while the current research is related to NADH regeneration during the Calvin cycle (artificial photosynthesis).

**Table 2:** Efficiency comparison of various methods/systems in the regeneration of enzymatically-active 1,4-NADH (highest efficiency at optimum conditions).

Electrode	Recovery of active NADH / %	References
**GC**	**97.45**	**Current work**
GC	98	[35]
GC-Ru	98	[31]
Au-Pt	64	[36]
Cu	54	[36]
Au	28	[36]
Au-Hg	10	[52]
Pt (non-modified)	50	[52]
Au-Hg/cholesterol	75	[52]
Pt/anion_charged_memb	65	[39]
Hg	50	[39]
Rh^3+^_PPY/C	52	[54]

## Conclusions

An electrochemical reactor was developed for the reduction of NAD^+^ to NADH during Calvin Cycle using a Glassy Carbon cathode operated at constant electrode potential. The results showed that GC electrode is highly efficient and electrocatalytically active in regenerating NADH from NAD^+^. At electrode potential of −2.30 V, a very high recovery of 97.45±0.8% was achieved. The reason for this high recovery was the formation of active hydrogen, H_ads_ on the electrode surface which is immediately available for the NAD-radical electrochemical hydrogenation. It was also shown that Tris-buffer solution resulted an improved conversion of NAD^+^ to the reduction products compared to phosphate buffer solution with slightly slower regeneration kinetics. The Tris-buffer concentration has no effect on the regeneration kinetics and recovery of NADH. In addition, temperature greatly affects the stability of regenerated NADH. Namely, at room temperature the NAD^+^/NADH was stable for a maximum period of 24 to 30 hr (4 cycles) while at low temperature it was stable for 96 hr. The developed system worked effectively to regenerate NADH in a real biochemical reactor in the presence of enzymes and ATP regeneration systems during the Calvin Cycle. However, the methodology for immobilization of the enzymes needs to be modified since it was not efficient as expected. Namely, the amount of capturing CO_2_ provided by the bicarbonate was really low due to significant decrease in efficiency of the enzymes after immobilization. By using CO_2_, this technology not only reduces greenhouse gas emissions, but it generates revenue by producing valuable and useful chemicals, including ethylene glycol. Therefore, our research will fit into advanced technology solutions and/or infrastructure for CO_2_ conversion into valuable, useful materials.

## Supporting information

S1 File Procedure for enzymatic assay. (DOCX)
