# Supplementary material for: Electrocatalytic CO2 fixation by regenerating reduced cofactor NADH during Calvin Cycle using glassy carbon electrode
Source: PLoS One. 2020 Sep 17;15(9):e0239340. doi: 10.1371/journal.pone.0239340 (PMC7497995; doi:10.1371/journal.pone.0239340)
Supplement: S1 File — (DOCX) [file pone.0239340.s001.docx]

**Supporting Information File**

**Electrocatalytic CO_2_ fixation by Regenerating Reduced Cofactor NADH during Calvin Cycle using Glassy Carbon Electrode**

Irshad Ali ^1^, Saeid Amiri ^2^, Nehar Ullah ^1^, Mohammad Younas ^1^, Mashallah Rezakazemi^3,*^

^1^ Department of Chemical Engineering, University of Engineering & Technology, Peshawar, Pakistan

^2^ Chemical & Petroleum Engineering Department, Sharif University of Technology, Tehran, Iran

^3^ Faculty of Chemical and Materials Engineering, Shahrood University of Technology, Shahrood, Iran

Corresponding author Email: m.younas@uetpeshawar.edu.pk, [mashalah.rezakazemi@gmail.com](mailto:mashalah.rezakazemi@gmail.com)

**SIF 1. Procedure for enzymatic assay**

To determine the enzymatic activity of the regenerated 1,4-NADH, activity tests were made according to the regular Sigma Quality Control Test Procedure (EC 1.8.1.4) which was further modified for this purpose using lipoamide dehydrogenase (5.3 U/mg, Calzyme laboratories, Inc. 153A0025) as an enzyme and DL-6,8-thioctic acid amide (Fluka T5875) as a substrate.

First, a volume of 0.2 mL of substrate and 0.1 mL of EDTA (Sigma ED4S) were added into 2.6 mL of regenerated 1,4-NADH in a 4 mL cuvette. The absorbance of the solution at 340 nm was monitored using a UV-Vis spectrophotometer, until reaching a steady state value. Then, 0.1 mL of the enzyme was injected into the cuvette while the absorbance was recorded until reaching a final constant value, signifying that the entire active 1,4-NADH formed during the electrolysis was consumed by the enzymatic reaction (Fig. SIF 1.1).

**Figure SIF1.1:** Time dependence of normalized absorbance (*A/A_0_*) of 1,4-NADH produced by electrolysis of a 1 mM NAD^+^ solution on a GC-Pt electrode at –1.6 V. *A_0_* is the absorbance value recorded before reaction (2) was initiated, while *A* is the absorbance recorded at any time after the initiation of reaction (2).

Finally, taking into account the initial and final absorbance at 340 nm, the purity of enzymatically-active 1,4-NADH produced by electrolysis was calculated (Eq. 1).

|  | Purity (recovery) of 1,4-NADH (%)= $\frac{\left[100-\left(A/A_{o}\right)\times100\right]}{\text{initial NAD}^{+}\text{ purity (\%)}}\times100\%$ | (1) |
| --- | --- | --- |

The procedure for the activity assay is described in detail below:

**Principle:**

|  |  | (2) |
| --- | --- | --- |

**Conditions:** *T* = 293 K, *pH* = 7.4, *A*_340nm_, light path *b* = 1cm

**Method:** UV/Vis spectrophotometer

**Reagents Preparation:**

**Reagent A**: 0.1 M phosphate buffer, pH 7.4 at 293 K

Prepare 150 mL by dissolving 2.04 g of potassium phosphate monobasic, anhydrous in deionized water. Adjust the pH to 7.4 with 1 N NaOH.

**Reagent B**: 0.028 M DL-6,8-thioctic acid amide

Prepare 2.5 mL by dissolving 0.01437 g of DL-6,8-thioctic acid amide in 1.5 mL ethanol (nondenatured). Dilute this solution with 1 mL of reagent A. Prepare fresh.

**Reagent C**: 0.3 M ethylenediaminetetraacetic acid (EDTA) with 2.0 % Albumin solution, pH 7.4.

Prepare 2.5 mL in deionized water using 0.22 g of EDTA and 0.0044 g of albumin bovine (Sigma A0281). Adjust the pH to 7.4 with 1 N NaOH.

**Reagent D:** Nicotinamide adenine dinucleotide, reduced form (NADH) produced by electrolysis of a 1 mM solution of NAD^+^ in phosphate buffer pH 7.4.

**Reagent E:** Lipoamide Dehydrogenase Enzyme Solution

Immediately before use, prepare a 10 mL solution of lipoamide dehydrogenase in cold reagent A by dissolving 0.0011 g.

**Table: SIF 1.1:** Pipette (in mL) the following reagents into suitable cuvettes.

| **Reagent** | **Reference 1** | **Reference 2** | **Test** |
| --- | --- | --- | --- |
| Reagent A | 2.6 | 2.6 | – |
| Reagent B | 0.2 | 0.2 | 0.2 |
| Reagent C | 0.1 | 0.1 | 0.1 |
| Reagent D | – | – | 2.6 |
| Reagent E | – | – | 0.1 |

First, zero the absorbance at 340 nm with Reference 1 and Reference 2, and then remove Reference 2 and replace it with “Test” (Table 1). Record the change in absorbance at 340 nm with time, until it becomes constant. Then add reagent E and record the absorbance with time until reaching a constant value.

The assay was first calibrated using commercially available NADH that contains 98% of enzymatically-active 1,4-NADH (Sigma N8129).

In order to determine the conversion of NAD^+^ to enzymatically‑active 1,4‑NADH, on different working electrodes, a UV/Vis calibration was curve developed as shown in Fig. SIF 1.2.

**Figure SIF 1.2:** Calibration curve for UV/Vis spectrometer.
